# Characterization of CRISPR-Cas Systems in *Shewanella algae* and *Shewanella haliotis*: Insights into the Adaptation and Survival of Marine Pathogens

**DOI:** 10.3390/pathogens13060439

**Published:** 2024-05-23

**Authors:** Jui-Hsing Wang, Po-Tsang Huang, Yao-Ting Huang, Yan-Chiao Mao, Chung-Hsu Lai, Ting-Kuang Yeh, Chien-Hao Tseng, Chih-Chuan Kao

**Affiliations:** 1 Division of Infectious Disease, Department of Internal Medicine, Taichung Tzu Chi Hospital, Buddhist Tzu Chi Medical Foundation, Taichung 427213, Taiwan; u702026@gmail.com; 2 Department of Internal Medicine, School of Medicine, Tzu Chi University, Hualien 970374, Taiwan; 3 Division of Pharmacy, Kaohsiung Armed Forces General Hospital, Kaohsiung 802301, Taiwan; a1018020014@mail.802.org.tw; 4 Department of Computer Science and Information Engineering, National Chung Cheng University, Chia-Yi 621301, Taiwan; ythuang@cs.ccu.edu.tw; 5 Division of Clinical Toxicology, Department of Emergency Medicine, Taichung Veterans General Hospital, Taichung 407219, Taiwan; doc1385e@gmail.com; 6 Division of Infectious Diseases, Department of Internal Medicine, E-Da Hospital, Kaohsiung 824005, Taiwan; laich6363@yahoo.com.tw; 7 School of Medicine, College of Medicine, I-Shou University, Kaohsiung 840301, Taiwan; 8 Division of Infectious Diseases, Department of Internal Medicine, Taichung Veterans General Hospital, Taichung 407219, Taiwan; tingkuangyeh@gmail.com; 9 Genomic Center for Infectious Diseases, Taichung Veterans General Hospital, Taichung 407219, Taiwan; 10 Division of Infectious Disease, Department of Internal Medicine, Tungs’ Taichung Metroharbor Hospital, Taichung 435403, Taiwan

**Keywords:** CRISPR–Cas, *Shewanella*, whole-genome sequencing

## Abstract

CRISPR-Cas systems are adaptive immune mechanisms present in most prokaryotes that play an important role in the adaptation of bacteria and archaea to new environments. *Shewanella algae* is a marine zoonotic pathogen with worldwide distribution, which accounts for the majority of clinical cases of *Shewanella* infections. However, the characterization of *Shewanella algae* CRISPR-Cas systems has not been well investigated yet. Through whole genome sequence analysis, we characterized the CRISPR-Cas systems in *S. algae*. Our results indicate that CRISPR-Cas systems are prevalent in *S. algae*, with the majority of strains containing the Type I-F system. This study provides new insights into the diversity and function of CRISPR-Cas systems in *S. algae* and highlights their potential role in the adaptation and survival of these marine pathogens.

## 1. Introduction

The CRISPR-Cas system (clustered regularly interspaced short palindromic repeats–CRISPR-associated protein) is a mechanism for natural immunity in bacteria, helping them to combat invading viruses (phages) by serving as adaptive immune systems that confer heritable resistance to foreign nucleic acids in most prokaryotes [1] and some viruses [2]. The CRISPR-Cas system stores and utilizes short DNA fragments that have been previously invaded by bacteriophages as immune memories, enabling bacteria to recognize and neutralize future invaders. This adaptive immune system allows bacteria to swiftly adapt to novel threats in their environment [3,4].

CRISPR-Cas systems have the ability to integrate segments of invading DNA as spacers into the CRISPR loci. Designed as a genome-editing tool, CRISPR–Cas systems require only a short RNA sequence and can recognize different target sequences [5]. Currently, CRISPR–Cas systems comprise two classes (class 1 and class 2) and are further classified into at least six different types [6]. According to the current classification, Class 1 encodes multiple Cas subunits to form a complex system, and Class 2 systems consist of a single multidomain protein and have been widely used as genome-editing tools, such as Cas9 and Cas12a [7,8].

*Shewanella* is a marine zoonotic pathogen with worldwide distribution. The predominant clinical infections it causes include blood stream infections from hepatobiliary sources and skin and soft tissue infections following seawater exposure [9,10]. 

Several highly diverse *Shewanella* species include *S. algae*, *S. haliotis*, *S. putrefaciens*, and *S. xiamenensis* [11,12]. *Shewanella algae* (the most common) and *S. haliotis* account for most clinical cases of *Shewanella* infections in the Western Pacific region [9,13]. Environment surveillance has also demonstrated these microorganisms are widespread in the marine environment, including water and aquaculture [14,15,16]. 

To date, a few genome analyses of the genus *Shewanella* have attempted to elucidate the association of genetic characteristics with potential pathogenic lineages [17,18,19]. There are two studies that have shown the presence of a CRISPR-Cas system in a single strain of *S. putrefaciens* [20] and *S. xiamenensis* [21]. However, the characterization of *Shewanella* using CRISPR-Cas systems has not yet been investigated, particularly for *S. algae*. 

In this article, we present a whole-genome sequence analysis of *S. algae* and *S. haliotis*. We used a combination of molecular biology techniques and bioinformatics to investigate the CRISPR-Cas systems in *S. algae* and *S. haliotis* and to identify candidate virulence genes. By using a multiple-database-based approach, we were able to cross-validate our results and achieve a comprehensive understanding of these adaptive immune systems in the marine zoonotic pathogens.

## 2. Materials and Methods

### 2.1. Shewanella Strains

A total of 17 *Shewanella* strains were included in the study (Table 1). These strains were collected from both clinical and environmental sources, and included ten *S. algae* and seven *S. haliotis* (labeld as *S. algae*/*S. haliotis*), a closely-related strain of *S. algae*. The seven strains were initially classified as *Shewanella haliotis* and later changed to *Shewanella algae* by NCBI due to the publication by Szeinbaum et al. [22]. All 11 clinical isolates are associated with invasive infection. The strains were preliminary identified using matrix-assisted laser desorption/ionization time-of-flight mass spectrometry (bioMérieux, Marcy-l′Étoile, France) and Sanger sequencing of the 16S rRNA gene [23]. The primers used for amplification of the 16S rRNA gene were B27F (5′-AGAGTTTGATCCTGGCTCAG-3′) and U1492R (5′-GGTTACCTTGTTACGACTT-3′). The PCR product was sequenced against the bacterial 16S rRNA gene sequences in the GenBank database of the National Center for Biotechnology Information using the BLASTn (optimized for Megablast) algorithm [24].

### 2.2. Whole-Genome Sequencing, Assembly, and Annotation

The strains were stored at −80 °C in Müller–Hinton broth containing 8.7% (*vol*/*vol*) glycerol until further usage. DNA was extracted from one colony of each strain grown on Trypticase Soy Agar with 5% sheep blood (Becton–Dickinson, Franklin Lakes, NJ, USA) using the QIAGEN Genomic-tip 100/G kit and the Genomic DNA Buffer Set (QIAGEN, Hilden, Germany) [25]. The DNA concentration was measured using a Qubit dsDNA HS Assay kit using Qubit 2.0 fluorometer (Life Technologies, Carlsbad, CA, USA). The genomic DNA was fragmented using Covaris S2 (Covaris, Woburn, MA, USA). The fragmented DNA was used to construct indexed PCR-free libraries using the multiplexed high-throughput sequencing TruSeq DNA Sample Preparation Kit (Illumina, San Diego, CA, USA), following the protocols provided by the manufacturer [26,27]. Whole-genome shotgun sequencing was performed using 2 × 250 bp paired-end sequencing on a MiSeq platform (Illumina, San Diego, CA, USA). 

The reads were filtered using duk (http://duk.sourceforge.net/ (accessed on 10 August 2023)) and trimmed with the FASTQX-toolkit fastqTrimmer (https://github.com/agordon/fastx_toolkit (accessed on 10 August 2023)). The reads were then assembled using Velvet v. 1.2.07 [28] and the resulting contigs were scaffolded using ALLPATHS v. R46652 [29]. The assembled DNA sequences of the studied strains were annotated using the National Center for Biotechnology Information (NCBI) Prokaryotic Genomes Automatic Annotation Pipeline (PGAAP). Functional classification of these annotated genes (e-value < 0.001) was carried out with the RPSBLAST v. 2.2.15 [30] in conjunction with the database of Clusters of Orthologous Groups of proteins (COGs). The sequencing data for this study have been uploaded in Supplementary Table S1 for reference.

### 2.3. CRISPR-Cas Systems Analysis

A multiple-database-based approach was used to analyze the CRISPR-Cas systems in the tested *Shewanella* strains. All predicated coding regions of the genome were subjected to analysis against CrisprCasFinder (https://crisprcas.i2bc.paris-saclay.fr/CrisprCasFinder/Index (accessed on 13 September 2023)) [31], CRISPRminer (http://www.microbiome-bigdata.com/CRISPRminer/ (accessed on 14 September 2023)) [32], and CRIS-PRcasIdentifier (https://github.com/BackofenLab/CRISPRcasIdentifier (accessed on 15 September 2023)) [33] to identify and cross-validate CRISPR-Cas types. These software and websites were used to provide consistent results, but each one also provided additional information. For example, CrisprCasFinder additionally provides CRISPR spacers and repeat consensus [34], and CRISPRminer provides Phage alignment results. 

In addition to identifying CRISPR-Cas systems, we also sought to identify candidate virulence genes in the *Shewanella* genomes. To facilitate this, we aligned the assembled genomes against the full Virulence Factors Database (VFDB) protein sequences dataset using BLASTX. We used the following criteria for identification: >45% identity; >450 bp aligned length; >95% alignment coverage; and E-value < 1 × 10^−45^. 

### 2.4. Nucleotide Sequence Accession Number

The sequencing data for each isolate were submitted to GenBank with BioProject record number PRJNA312015.

## 3. Results 

**Figure 1:**
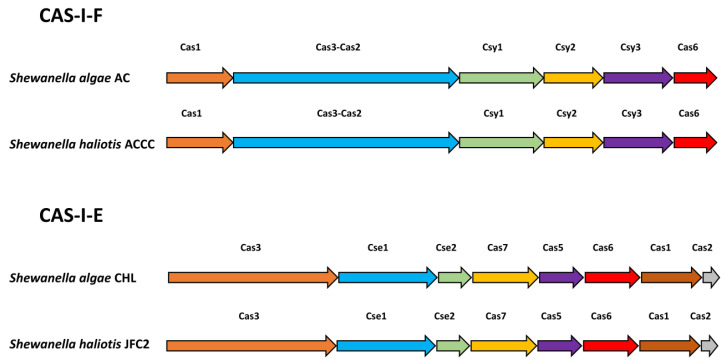
Overview of *Shewanella algae* CRISPR-Cas structures.

CRISPR-Cas systems were detected in all 17 strains in this study (Table 2). Analysis of the *cas* gene structures revealed that eight *S. algae* and five *S. algae*/*S. haliotis* genomes possessed a typical I-F cas structure, including the universal *csy1*, *csy2*, *csy3*, and *cas6f* genes as well as the type I signature genes *cas3*, which is fused to *cas2*, a unique feature of I-F-type systems. Furthermore, we identified both CAS-I-E and CAS-I-F in two closely related strains of *Shewanella*. The alignment results demonstrated the consistency in the distribution of cas genes for each Cas type. Two *S. algae* and two *S. algae*/*S. haliotis* genomes showed a type I-E structure, excluding the *cas4* gene (Figure 1). All of the CRISPR-positive strains possessed only one valid CRISPR-Cas structure. The spacer counts ranged from 10 to 86 for type I-F and from 28 to 110 for type I-E systems. The CRISPR repeat sequences for each type are similar (Table 3). 

Our analysis also revealed that 20 of the identified spacers had putative origins from phages. Most of these came from phages detected in *Vibrio* (Table 4). By aligning ORF-encoded protein sequences with the virulence factor database, we screened the *Shewanella* genome for putative virulence-associated genes. The genomic analysis revealed strain-specific gene *tapW* which is associated with twitching ATPase. *S. algae* also harbors *exeG* and *gspG*, which are associated with general secretion pathway protein G (Supplementary Table S2).

## 4. Discussion

In this study, we aimed to conduct a sequence analysis and characterization of the CRISPR-Cas system in *S. algae* and *S. haliotis*. Type I-F and type I-E CRISPR-Cas systems were identified in all of the *Shewanella* strains analyzed in our study. The majority of clinical isolates contained a type I-F system. The type I-F and type I-E CRISPR-Cas systems were hypothesized to comprise a bacterial defense mechanism against phage and other genetic elements [4]. Previous studies have reported the presence of type I CRISPR-Cas systems in other species of *Shewanella*, including type I-F in *S. putrefaciens* [20] and type I-E in *S. xiamenensis* [21]. A further comparative genome analysis of 41 genomes supported these previous reports; the I-F type was reported to be the most frequent system (27/41; 65.85%) in *S. algae*, and *S. putrefaciens* and the type I-E system was found in *S. xiamenensis* strains (21.9%) [19].

Regarding the CRISPR arrays associated with each cas operon, our data show that the cas operons are well conserved in tested strains, displaying two distinct cas gene profiles. Cas operons are clusters of genes found in some bacteria that contain genes related to the CRISPR-Cas system. These gene clusters may help maintain the host’s defenses’ functionality and were found to be relevant in a recent study [19]. On the other hand, we also observed diverse spacers, suggesting a high diversity of phage environments and the potential immune function of the CRISPR-Cas system in *Shewanella*. *S. algae* is widely distributed in marine and freshwater habitats. An early study showed various spacers in different *S. xiamenensis* strains among CRISPR-Cas systems within changing environments [35]. 

Our study complements these findings and expands our understanding of CRISPR-Cas systems in *S. algae*. The development of the CRISPR field has led to many clinical and biological implications [36,37]. Genome editing based on the CRISPR-Cas system enables efficient and affordable gene engineering [38]. Active CRISPR systems allow for the transcription and processing of the CRISPR arrays into short CRISPR RNAs (crRNAs) that contain a spacer and are covalently linked to the Cas endonuclease [39]. When the cell is re-infected, this complex utilizes crRNA base complementarity to recognize and degrade DNA or RNA from elements containing the spacer sequence [40]. 

Previous research has shown that the spacers present in the CRISPR arrays of bacteria are acquired from phages that have invaded the bacteria [41]. This means that the CRISPR-Cas systems of bacteria maintain a record, or molecular memory, of previous infections. This is also the mechanism by which these systems confer adaptive immunity to bacteria and archaea against foreign DNA. Additionally, the presence of these spacers can also be used to trace the path of certain prokaryotic pathogens, as the spacers provide a record of the phages that have been present in a particular strain’s environment [42]. The fact that the spacers have originated from phages is crucial in understanding the biological records of past phage–bacteria interactions. Spacers can be utilized to predict hosts of unknown phages, providing insights into the history of interactions between phages and bacteria [43]. When studying the resistance of *Shewanella* spp., the number and types of spacer sequences in CRISPR also play an important role. One study showed that CRISPR/Cas9 genome editing technology can reverse the resistance of *S. algae* to carbapenem antibiotics [38]. The latest study indicated that spacer sequences are crucial components of the CRISPR system [35]. Different numbers and types of spacer sequences within various *Shewanella*-specific CRISPR systems demonstrate their significant role in the CRISPR-Cas system [35,44]. In general, *S. algae* were highly susceptible to aminoglycosides and carbapenems but were generally resistant to penicillin [45,46]. Reports of carbapenem and colistin resistance in *S. algae* have been increasing [38]. Therefore, it has been proposed as a potential source of antibiotic resistance in marine environments and deserves special attention in healthcare facilities.

Our findings of diverse spacers in *S. algae* and *S. haliotis* suggest a high diversity of phage environments and potential immune functions of CRISPR-Cas systems in these marine zoonotic pathogens. Other studies have also reported similar findings [35,47]. The bacterial resistance to phages and the activity of CRISPR was demonstrated to be correlated with CRISPR spacers diversity [48,49]. Previous spatially diverse models suggested that the CRISPR array evolved to have between 20 and 30 spacers [50], which is lower than our observation in *Shewanella*. Our results are also consistent with a coevolutionary model which suggested that the high diversity of spacers in these strains may have evolved in response to a diverse phage environment. These findings are in parallel with empirical observations of the adaptive immune system in bacteria [51]. The mechanisms of *Shewanella* immune system and the implications warrant further study [52]. 

Our study suggests that CRISPR-Cas systems are prevalent in *S. algae* and *S. haliotis* may play a role in their adaptation to new environments. The study further suggested a complex trade-off between gains and losses in CRISPR-Cas systems when adapting to new environments. There is growing evidence that CRISPR-Cas systems constrain horizontal gene transfer [53]. On the other hand, the elimination of immunity to newly acquired mobile elements that confer a fitness benefit could lead to the loss of CRISPR-Cas systems [54]. 

Further research is needed to determine the genetic diversity, pathogenicity, antibiotic resistance, and specific mechanisms of *Shewanella*’s immune system and the implications of these findings for understanding the ecology and pathogenesis of these organisms. Overall, our study expands our understanding of the diversity and function of CRISPR-Cas systems in *Shewanella* and creates new opportunities for investigating the role of these systems in the adaptation and survival of these pathogens in the marine environment.

## 5. Conclusions

In summary, our study found that CRISPR-Cas systems are prevalent in *S. algae* and *S. haliotis*, with most strains possessing a type I-F CRISPR-Cas system. Additionally, we identified a unique feature of a fused cas3 and cas2 gene in type I-F systems, and also identified different CRISPR-Cas systems in two different species of *Shewanella*. Furthermore, we found that most spacers had putative origins from phages and identified virulence-associated genes in the *Shewanella* genome. These findings provide new insights into the CRISPR-Cas systems in these marine zoonotic pathogens and the potential for these systems to play a role in their adaptation to new environments.

## Figures and Tables

**Table 1:** *Shewanella* strains in this study.

Species	Strain	Geography	Source
*S. algae*	AC	Taiwan	*Haliotis diversicolor*
*S. algae*	CHL	Taiwan	bile
*S. algae*	CLS1	Taiwan	wound
*S. algae*	CLS2	Taiwan	blood
*S. algae*	CLS3	Taiwan	blood
*S. algae*	CLS4	Taiwan	blood
*S. algae*	melkephyllucas	Taiwan	blood
*S. algae*	SYT2	Taiwan	*Crassostrea gigas*
*S. algae*	SYT3	Taiwan	ocean water
*S. algae*	YHL	Taiwan	wound
*S. algae*/*S. haliotis*	ACCC	Taiwan	bile
*S. algae*/*S. haliotis*	JFC2	Taiwan	*Crassostrea gigas*
*S. algae*/*S. haliotis*	JFC3	Taiwan	*Crassostrea gigas*
*S. algae*/*S. haliotis*	JFL	Taiwan	blood
*S. algae*/*S. haliotis*	MSW	Taiwan	*Meretrix lusoria*
*S. algae*/*S. haliotis*	RC	Taiwan	blood
*S. algae*/*S. haliotis*	YTH	Taiwan	blood

**Table 2:** Spacers and *cas* genes found in CRISPR-system in the study.

Organisms		Origin and Spacer Index		
Species	Strain	Cas Type	Spacer	Cas Genes
*S. algae*	AC	CAS-I-F	63	Cas6, Csy3, Csy2, Csy1, Cas3-Cas2, Cas1
*S. algae*	CHL	CAS-I-E	110	Cas2, Cas1, Cas6, Cas5, Cas7, Cse2, Cse1, Cas3
*S. algae*	CLS1	CAS-I-F	51	Cas1, Cas3-Cas2, Cas6, Csy1, Csy2, Csy3
*S. algae*	CLS2	CAS-I-F	10	Cas1, Cas3-Cas2, Cas6, Csy1, Csy2, Csy3
*S. algae*	CLS3	CAS-I-F	86	Cas1, Cas3-Cas2, Cas6, Csy1, Csy2, Csy3
*S. algae*	CLS4	CAS-I-F	43	Cas6, Csy3, Csy2, Csy1, Cas3-Cas2, Cas1
*S. algae*	melkephyllucas	CAS-I-F	45	Cas6, Csy3, Csy2, Csy1, Cas3-Cas2, Cas1
*S. algae*	SYT2	CAS-I-E	64	Cas2, Cas1, Cas6, Cas5, Cas7, Cse2, Cse1, Cas3
*S. algae*	SYT3	CAS-I-F	24	Cas1, Cas3-Cas2, Cas6, Csy2, Csy3
*S. algae*	YHL	CAS-I-F	43	Cas6, Csy3, Csy2, Csy1, Cas3-Cas2, Cas1
*S. algae*/*S. haliotis*	ACCC	CAS-I-F	47	Cas1, Cas3-Cas2, Csy1, Csy2, Csy3, Cas6
*S. algae*/*S. haliotis*	JFC2	CAS-I-E	28	Cas3, Cse1, Cse2, Cas7, Cas5, Cas6, Cas1, Cas2
*S. algae*/*S. haliotis*	JFC3	CAS-I-F	31	Cas6, Csy3, Csy2, Csy1, Cas3-Cas2, Cas1
*S. algae*/*S. haliotis*	JFL	CAS-I-E	66	Cas3, Cse1, Cse2, Cas7, Cas5, Cas6, Cas1, Cas2
*S. algae*/*S. haliotis*	MSW	CAS-I-F	44	Cas1, Cas3-Cas2, Csy1, Csy2, Csy3, Cas6
*S. algae*/*S. haliotis*	RC	CAS-I-F	27	Cas1, Cas3-Cas2, Csy1, Csy2, Csy3, Cas6
*S. algae*/*S. haliotis*	YTH	CAS-I-F	27	Cas1, Cas3-Cas2, Csy1, Csy2, Csy3, Cas6

**Table 3:** Repeat consensus of *Shewanella* strains in the study.

Species	Strain	Accession No.	Repeat Consensus
*S. algae*	AC	SAMN04492223	TTTCTAAGCTGCCTGGGCGGCAGTGAAC
*S. algae*	CHL	SAMN04492221	CGGTTTATCCCCGTGGGTGCGGGGAACAC
*S. algae*	CLS1	SAMN04492203	GTTCACTGCCGCCCAGGCAGCTTAGAAA
*S. algae*	CLS2	SAMN04492207	TTTCTAAGCTGCCTGGGCGGCAGTGAAC
*S. algae*	CLS3	SAMN04492209	TTTCTAAGCTGCCTGGGCGGCAGTGAAC
*S. algae*	CLS4	SAMN04492210	TTTCTAAGCTGCCTGGGCGGCAGTGAAC
*S. algae*	melkephyllucas	SAMN04492222	TTTCTAAGCTGCCTGGGCGGCAGTGAAC
*S. algae*	SYT2	SAMN04492205	CGGTTTATCCCCGTGGGTGCGGGGAACTC
*S. algae*	SYT3	SAMN04492208	GTTCACTGCCGCCCAGGCAGCTTAGAAA
*S. algae*	YHL	SAMN04492206	TTTCTAAGCTGCCTGGGCGGCAGTGAAC
*S. algae*/*S. haliotis*	ACCC	SAMN04492216	GTTCACTGCCGCCCAGGCAGCTTAGAAA
*S. algae*/*S. haliotis*	JFC2	SAMN04492214	GAGTTCCCCGCACCCACGGGGATAAACCG
*S. algae*/*S. haliotis*	JFC3	SAMN04492215	TTTCTAAGCTGCCTGGGCGGCAGTGAAC
*S. algae*/*S. haliotis*	JFL	SAMN04492224	GTGTTCCCCGCACCCACGGGGATAAACCG
*S. algae*/*S. haliotis*	MSW	SAMN04492233	GTTCACTGCCGCCCAGGCAGCTTAGAAA
*S. algae*/*S. haliotis*	RC	SAMN04492217	GTTCACTGCCGCCCAGGCAGCTTAGAAA
*S. algae*/*S. haliotis*	YTH	SAMN04492218	GTTCACTGCCGCCCAGGCAGCTTAGAAA

**Table 4:** Phage-originated spacers in *Shewanella algae*.

Species	Strain	Phage ID	Phage Name	Identity
*S. algae*	CHL	ref|NC_015465.1|	Synechococcus phage S-CBS3	100
		ref|NC_016164.1|	Synechococcus phage S-CBS1	100
*S. algae*	CLS1	ref|NC_004456.1|	Vibrio phage VHML	93.75
		ref|NC_027981.1|	Vibrio phage VP585	96.88
*S. algae*	CLS3	ref|NC_004456.1|	Vibrio phage VHML	93.75
		ref|NC_004456.1|	Vibrio phage VHML	100
		ref|NC_004456.1|	Vibrio phage VHML	100
		ref|NC_004456.1|	Vibrio phage VHML	96.88
		ref|NC_009016.1|	Vibrio phage VP882	100
		ref|NC_009016.1|	Vibrio phage VP882	100
		ref|NC_019722.1|	Vibrio phage vB_VpaM_MAR	100
		ref|NC_019722.1|	Vibrio phage vB_VpaM_MAR	100
		ref|NC_027981.1|	Vibrio phage VP585	96.88
		ref|NC_027981.1|	Vibrio phage VP585	93.75
*S. algae*	YHL	ref|NC_004456.1|	Vibrio phage VHML	100
		ref|NC_029094.1|	Pseudoalteromonas phage H101	100
*S. algae*/*S. haliotis*	MSW	ref|NC_004456.1|	Vibrio phage VHML	100
		ref|NC_005887.1|	Burkholderia virus BcepC6B	95.83
		ref|NC_019722.1|	Vibrio phage vB_VpaM_MAR	100
		ref|NC_027981.1|	Vibrio phage VP585	96.88

## Data Availability

The sequencing data for each isolate were submitted to GenBank with BioProject accession number PRJNA312015.

## Supplementary Materials

The following supporting information can be downloaded at: https://www.mdpi.com/article/10.3390/pathogens13060439/s1, Table S1: Summary of sequencing and assembly data; Table S2: Virulence genes identified in the *Shewanella*.
